# Unraveling the rapid radiation of crested newts (*Triturus cristatus *superspecies) using complete mitogenomic sequences

**DOI:** 10.1186/1471-2148-11-162

**Published:** 2011-06-14

**Authors:** Ben Wielstra, Jan W Arntzen

**Affiliations:** 1 Netherlands Centre for Biodiversity - NCB Naturalis, P. O. Box 9517, 2300 RA Leiden, The Netherlands; 2 International Institute for Geo-Information Science and Earth Observation - ITC, P.O. Box 6, 7500 AA, Enschede, The Netherlands

## Abstract

**Background:**

The rapid radiation of crested newts (*Triturus cristatus *superspecies) comprises four morphotypes: 1) the *T. karelinii *group, 2) *T. carnifex *- *T. macedonicus*, 3) *T. cristatus *and 4) *T. dobrogicus*. These vary in body build and the number of rib-bearing pre-sacral vertebrae (NRBV). The phylogenetic relationships of the morphotypes have not yet been settled, despite several previous attempts, employing a variety of molecular markers. We here resolve the crested newt phylogeny by using complete mitochondrial genome sequences.

**Results:**

Bayesian inference based on the mitogenomic data yields a fully bifurcating, significantly supported tree, though Maximum Likelihood inference yields low support values. The internal branches connecting the morphotypes are short relative to the terminal branches. Seen from the root of *Triturus *(NRBV = 13), a basal dichotomy separates the *T. karelinii *group (NRBV = 13) from the remaining crested newts. The next split divides the latter assortment into *T. carnifex *- *T. macedonicus *(NRBV = 14) versus *T. cristatus *(NRBV = 15) and *T. dobrogicus *(NRBV = 16 or 17).

**Conclusions:**

We argue that the Bayesian full mitochondrial DNA phylogeny is superior to previous attempts aiming to recover the crested newt species tree. Furthermore, our new phylogeny involves a maximally parsimonious interpretation of NRBV evolution. Calibrating the phylogeny allows us to evaluate potential drivers for crested newt cladogenesis. The split between the *T. karelinii *group and the three other morphotypes, at ca. 10.4 Ma, is associated with the separation of the Balkan and Anatolian landmasses (12-9 Ma). No currently known vicariant events can be ascribed to the other two splits, first at ca. 9.3 Ma, separating *T. carnifex *- *T. macedonicus*, and second at ca. 8.8 Ma, splitting *T. cristatus *and *T. dobrogicus*. The crested newt morphotypes differ in the duration of their annual aquatic period. We speculate on the role that this ecological differentiation could have played during speciation.

## Background

**Figure 1:**
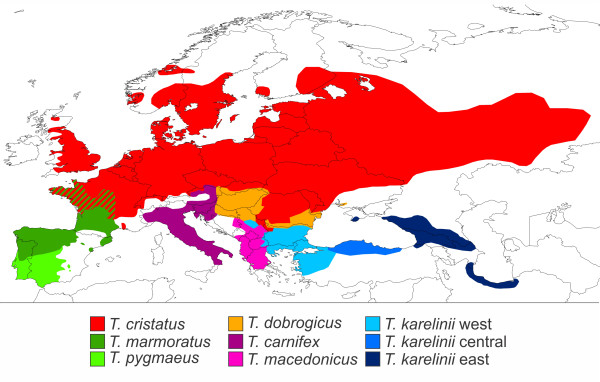
**The distribution of the genus *Triturus***. Shown are the ranges for all the different species; the range of the *T. karelinii *group is partitioned according to the three distinct mitochondrial DNA clades the group is composed of (cf. [16]). Note the partially overlapping ranges of the crested newt *T. cristatus *and the marbled newt *T. marmoratus*. The map is based on [6] and updated following recent findings.

Understanding the temporal framework in which species have originated is fundamental to historical biogeography and evolutionary studies. However, obtaining a reliable phylogeny for a model system is not always straightforward. Rapid radiations are notoriously difficult in this respect, and the older the radiation, the more pronounced the problem will be [1]. The crested newt *Triturus cristatus *superspecies (Amphibia: Salamandridae), distributed in Europe and adjacent Asia (Figure 1), is an example of a relatively old, rapid radiation, for which it has proved problematic to obtain a resolved phylogenetic tree.

**Figure 2:**
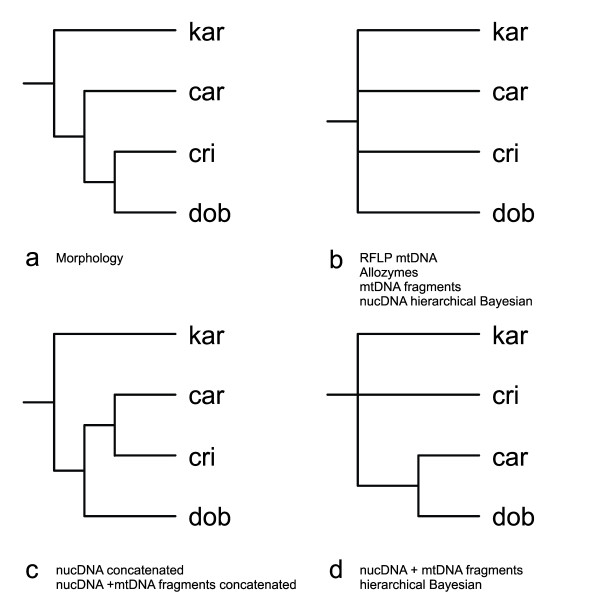
**A summary of the different phylogenetic hypotheses for the four crested newt morphotypes as suggested by previous studies**. The datasets supporting each tree are noted below it (see the main text for details and references). Abbreviations used for the four morphotypes are: kar = *T. karelinii *group; car = *T. carnifex *- *T. macedonicus*; cri = *T. cristatus*; dob = *T. dobrogicus*.

The crested newt superspecies encompasses four morphological groups, hereafter referred to as 'morphotypes'. Ordered from a stocky build with sturdy limbs, to slender with small limbs, via two intermediate stages, these are: 1) the *T. karelinii *group, 2) *T. carnifex *- *T. macedonicus*, 3) *T. cristatus *and 4) *T. dobrogicus*. The morphotypes are characterized by discrete differences in the number of rib-bearing pre-sacral vertebrae (NRBV) [2]. The typical NRBV count is 13 for the *T. karelinii *group, 14 for *T. carnifex *- *T. macedonicus*, 15 for *T. cristatus *and 16 or 17 for *T. dobrogicus*. The marbled newts, *T. marmoratus *and *T. pygmaeus*, which make up the crested newts' sister group, have the heaviest body build in the genus and possess a typical NRBV count of 12. Over ninety per cent of these newts can be correctly identified based on NRBV counts alone; interspecific hybridization along parapatric contact zones (Figure 1) is suggested to account for most of the remaining intraspecific variation [2-4]. Crested newt morphology has been interpreted as reflecting phylogeny [2]. A maximally parsimonious interpretation of NRBV (interpreting the ancestral crested newt body shape as relatively robust and the more slender body shapes as derived) suggests a branching order as shown in Figure 2a.

With the advent of molecular techniques, independent data became available and multiple molecular markers have now been employed to attempt to resolve the crested newt phylogeny. Based on restriction fragment length polymorphism of the mitochondrial genome (employing eleven restriction enzymes), a polytomy was found (Figure 2b) [5]. Similarly, analysis of a suite of forty enzyme loci and 642 bp of mitochondrial sequence data resulted in a polytomous relationship for the four morphotypes (Figure 2b) [6]. A polytomy could simply reflect a lack of phylogenetic resolution in the data. However, as the different datasets both pointed towards a polytomy, it was suggested that the four crested newt morphotypes truly split practically simultaneously [6].

A later effort using sequence data of five nuclear DNA (2589 bp) and two mitochondrial DNA markers (1747 bp) revealed a more varied picture [7]. An analysis using the sequence data concatenated found a fully bifurcating and significantly supported phylogeny (Figure 2c). There are, however, theoretical objections to data concatenation, as this method does not consider the unique topological history that each individual gene possesses [8]. Therefore, phylogenetic inference was also carried out using a hierarchical Bayesian analysis, which does explicitly take the effects of gene tree heterogeneity into account. This analysis indeed produced different results. Based on the five nuclear DNA markers, a fourfold polytomy was found again (Figure 2b). This fourfold polytomy could be expanded to a trichotomy by incorporating the mitochondrial DNA data, but the single sister relationship found was incongruent with the data concatenation approach (Figure 2d).

Previous molecular studies have firmly established that the crested newt radiation occurred in a brief time interval. However, they yielded conflicting phylogenetic hypotheses and have been unable to settle the relationship among the morphotypes. Furthermore, all phylogenetic hypotheses found so far are in conflict with the tree suggested by morphology. In this study we further explore the crested newt phylogeny, this time employing complete mitogenomic sequences. As the mitochondrial genome contains tenfold the bp studied up to now (~17 Kbp vs. ~1.7 Kbp), it is a promising source of phylogenetic resolution (cf. [9]). We here analyse nine newly-sequenced mitogenomes, representing all *Triturus *species, and manage to obtain a fully-resolved crested newt phylogeny. We discuss this new phylogeny with respect to previous attempts to obtain the *Triturus *tree and speculate on causes for cladogenesis.

## Results

**Figure 3:**
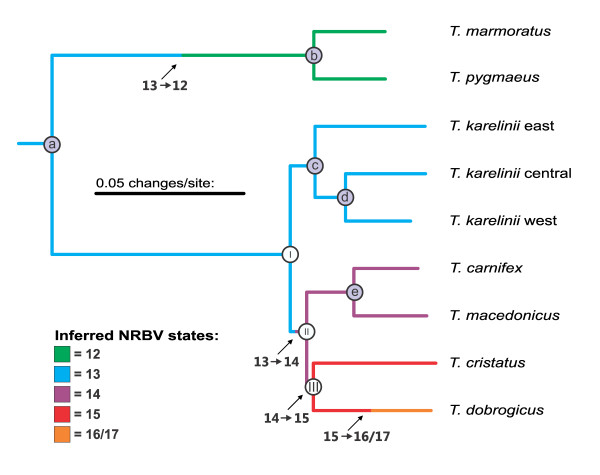
**The mitogenomic *Triturus *tree resulting from the Bayesian inference**. The *Calotriton asper *outgroup is not shown. Nodes are coded and correspond to table 2; the nodes of interest, separating the crested newt morphotypes, are coded I-III (the remaining nodes are coded a-e). The NRBV additions required to explain the NRBV variation observed in *Triturus *today are noted along the phylogeny (interpreting NRBV = 13 as the ancestral character state, see Additional File 1). The exact timing of inferred NRBV shifts is not known, only that they are positioned on a particular branch.

We present a mitogenomic *Triturus *phylogeny (Figure 3) based on a division of the mitogenomic sequence data into 42 data partitions, as this partitioning strategy is preferred over the simpler ones tested (Table 1). Bayesian inference identifies a basal dichotomy in the crested newt superspecies between the *T. karelinii *group and the other morphotypes (node I in Figure 3). The next bifurcation divides the latter assortment in *T. carnifex *- *T. macedonicus *(node II) versus *T. cristatus *and *T. dobrogicus *(node III). Posterior probabilities are ≥ 0.95 (Table 2). Although Maximum Likelihood inference yields the same branching order and similar branch lengths (tree not shown), the bootstrap support values for two of the three nodes associated with radiation of crested newt morphotypes (nodes II and III) are low (Table 2). The mitogenomic phylogeny is characterized by long terminal branches, which are connected by short internal branches. The three nodes connecting the crested newt morphotypes represent a narrow time window (approximately 10.4-8.8 Ma) and have small confidence intervals, independent of dating method used (Table 2). Three character state transitions are required to explain the NRBV differentiation across the four crested newt morphotypes, two of which are situated on short internal branches (Figure 3).

**Table 1:** Evaluation of the optimal partitioning scheme for the mitogenomic sequence data based on Bayes factor analysis

	42	6	29	16
16	5,300.00	4,554.12	4,553.18	-
29	746.82	0.94	-	-
6	745.89	-	-	-
42	-	-	-	-

A pairwise comparison of the four tested partitioning strategies (6, 16, 29 or 42 data partitions), which are ordered from highest to lowest marginal likelihood from left to right and vice versa from top to bottom. The marginal likelihoods, in order from highest to lowest, are: 42 = -48316.02, 6 = -48688.96, 29 = -48689.43 and 16 = -50966.02.

**Table 2:** Support values and temporal estimates for *Triturus *nodes

	Support values		Dating r8s		Dating BEAST	
Node	MrBayes	RAxML	Mean	95% CI	Mean	95% CI
I	1.0	100	10.3	9.4-11.2	10.4	9.4-11.5
II	1.0	62	9.2	8.5-9.4	9.3	8.3-10.2
III	0.95	46	8.7	8.0-9.4	8.8	7.8-9.7
a #	1.0	100	28.0	24.9-31.1	27.6	24.8-30.8
b	1.0	100	5.9	5.0-6.8	5.6	4.7-6.6
c	1.0	100	8.3	7.3-9.3	8.3	7.3-9.4
d	1.0	100	5.8	5.1-6.6	5.8	5.0-6.7
e #	1.0	100	5.33	n/a	5.33	n/a

The coded nodes correspond to Figure 2. The nodes of interest, separating the crested newt morphotypes, are coded I-III (the remaining nodes are coded a-e). Nodes marked with a # are used for temporal calibration.

## Discussion

### The mitogenomic *Triturus *tree

The full mitogenomic dataset has provided the phylogenetic resolution that the crested newt case required. Previous approaches using only part of the mitochondrial genome found a polytomy for the four morphotypes, but based on the full mitochondrial DNA sequences we managed to resolve this polytomy. Under Bayesian inference, we find a fully bifurcating phylogeny, with significant support for the three nodes connecting the four crested newt morphotypes (i.e. nodes I-III in Figure 3 and Table 2). In comparison, Maximum Likelihood bootstrapping finds equivocal results for two of the three nodes (nodes II and III). Disparity in support values between both methods is known to occur at short internodes, where Bayesian inference appears to better exploit the relatively small number of informative characters [10]. Our confidence in the Bayesian phylogeny is increased by its correspondence to the branching order suggested by a maximally parsimonious interpretation of NRBV evolution (Figure 2a and 3).

The mitochondrial genome, given its non-recombining nature, behaves as a single gene and, due to stochasticity in the coalescent process, the branching order it suggests is not necessarily congruent with the true species tree [11]. The motivation behind studying independent gene trees (i.e. multiple nuclear genetic markers) is that these should ultimately converge upon the overarching species tree [8]. However, a recent hierarchical Bayesian analysis based on five nuclear markers did not yield a resolved crested newt phylogeny [7]. This lack of resolution could be explained by the rapidness of the radiation of the crested newt morphotypes, as repeated cladogenesis within the temporal domain of the lineage sorting process increases the chance of a mismatch between gene trees and species tree [1,8,11-13]. Such a risk of gene tree - species tree discordance is smaller for the mitochondrial genome, because lineage sorting is realized faster compared to the nuclear genome (given the fourfold smaller effective population size of the mitochondrial genome due to haploid and uniparental inheritance [14,15]).

We do not claim that our current attempt resolves the crested newt species tree once and for all; studying a much larger battery of nuclear DNA markers than previously used is required to further home in on the crested newt species tree [12]. However, considering the data currently available, we suggest that the Bayesian mitogenomic phylogeny as yet provides the most reliable estimation of the crested newt species tree. We here employ the new phylogeny to explore the potential causes underlying the splits between the crested newt morphotypes.

### The potential of paleogeography to explain crested newt speciation

Based on temporal estimates associated with the crested newt splits (Table 2), potential vicariant events can be identified by consulting paleogeographic reconstructions. We here concentrate on the three splits which gave rise to the four crested newt morphotypes (i.e. nodes I-III in Figure 3); for vicariant events underlying the three splits within morphotypes (i.e. nodes c-e in Figure 3), see [6,16]. An earlier attempt to reconstruct the historical biogeography of crested newts assumed a 'hard' polytomous relationship for the four morphotypes [6]. In effect, a temporal estimate was only provided for the crown of the crested newts and proposed vicariant events were derived from this date.

The present study has resolved the relationships among the morphotypes (i.e. the polytomy in [6] turned out to be 'soft' after all). In line with the increased phylogenetic resolution, more recent dates are appointed to the newly resolved nodes and their morphotype lineages. The crested newt crown, i.e. the split between the *T. karelinii *group and the remaining crested newts (node I in Figure 3), is dated at ca. 10.4 Ma. The origin of the Aegean Sea at 12-9 Ma [17], which separated the Balkan Peninsula from Anatolia, is a likely underlying vicariant event (sensu [6]). No obvious vicariant events can be associated with the two splits that gave rise to the three remaining morphotypes [18]: the separation of *T. carnifex *- *T. macedonicus *versus *T. cristatus *and *T. dobrogicus *(node II) at approximately 9.3 Ma and the split between *T. cristatus *and *T. dobrogicus *(node III) around 8.8 Ma (contra [6]).

No comprehensive paleogeographical reconstructions are as yet available for the Balkan Peninsula in the period between 11 and 8.5 Ma [18]. It is feasible that vicariant events relevant to the crested newt case have yet to be discovered. As a way forward, we suggest that more taxa with Balkan distributions should be surveyed in a historical biogeographical context [6,19]. By uncovering congruent spatio-temporal signatures, such studies should assist in paleogeographical reconstruction of the Balkan Peninsula. This strategy might not be so straightforward; genetic structuring in the two other groups of newts that occur on the Balkan Peninsula (*Ichthyosaura *and *Lissotriton*), though relatively old, originated considerably more recent than that in the crested newts [20,21].

### Could ecological divergence have played a role in crested newt speciation?

The role of ecological divergence in historical biogeography is often regarded as passive; external factors such as geology and climate are considered to be responsible for the actual dividing of ancestral stocks and potential ecological divergence occurs at a later point [22]. However, ecology can play an active role in the speciation process: disruptive selection along an ecological gradient can result in restrictions to gene flow, in the absence of geographical isolation [22-25]. Could such a parapatric mode of speciation apply to the crested newt case?

The crested newt morphotypes do show ecological differentiation in the time they annually spend in the water. The duration of the annual aquatic period is three months in the *T. karelinii *group, four in *T. carnifex*, five in *T. cristatus *and six in *T. dobrogicus *[4]. Crested newts thus show a correlation between phenotype and phenology: sturdy bodies and a low NRBV count are associated with a more terrestrial way of life and slender bodies and a high NRBV count with a more aquatic life style [4]. This notion of a terrestrial versus aquatic trade-off associated with body shape is further supported by the even more robust marbled newt *T. marmoratus*, whose two-month aquatic phase is the shortest of the *Triturus *newts [6].

The body shape differentiation in crested newts occurred over a brief timespan (Figure 3 and Table 2): at least two of the three NRBV additions during crested newt evolution are associated with short internal branches. Under the assumption that today's phenology-phenotype correlation has been valid through time, the ecological divergence of the crested newt morphotypes must have similarly taken place over a short period. Considering the fundamental role water bodies play in amphibian reproduction [26], it is reasonable to suggest that differences in the availability of standing water would present different adaptive peaks. The time frame of the crested newt radiation corresponds to a period of increased seasonality in Eurasia during the Late Miocene (11.6-5.3 Ma), associated with the uplift of the Tibetan Plateau, which led to a more heterogeneous landscape in terms of humidity [27].

With a clear speciation scenario involving vicariance lacking, we, as an alternative hypothesis, suggest that the crested newts' body shape differentiation reflects a rapid adaptive radiation to different water regimes. It should be noted that these two hypotheses are not necessarily mutually exclusive.

### Reflections on NRBV and its adaptive value in *Triturus*

Salamanders have become a model group to understand patterns of morphological evolution [28]. Variation of salamander body shape has been accomplished by modifying the vertebral column, by either altering the length of the individual vertebrae or by changing the total number of vertebrae [29]. Interestingly, the intrageneric range in NRBV count shown by *Triturus *is unprecedented in Salamandridae (see Additional file 1), suggesting body shape plasticity played a prominent role during crested newt evolution. Work on the genetic pathways underlying the evolutionary development of the different *Triturus *morphotypes will provide more insight into this phenomenon (J.M. Ziermann et al., in prep.).

What could be the adaptive value of the crested newt NRBV radiation? The dualism of an amphibian lifestyle poses conflicting demands on body shape. The rapid adaptive radiation scenario we propose for the crested newts, suggests that the balance struck for this trade-off differs among the morphotypes, due to the different ecological background each of them experiences. What do we currently understand about the differential performance of the morphotypes in the aquatic and the terrestrial environment? Although [30] found that *Triturus *stockiness is positively correlated with running speed, this is not so for slenderness and swimming speed. What could be the reason for this partially unexpected result? Maybe 'speed' is not the most suitable way to characterize terrestrial or aquatic specialization? Perhaps body elongation benefits more aquatic *Triturus *newts some other way, e.g. by increasing maneuverability or by providing more space for egg production [30]? More research is required to elucidate the adaptive value of the different *Triturus *body shapes against distinct ecological backgrounds.

## Conclusions

Although it has proven difficult to resolve the rapid radiation of crested newts, by employing full mitochondrial DNA sequence data we now have a precise estimate of the chronology of branching events. The relationship among the four crested newt morphotypes found agrees with a maximally parsimonious interpretation of NRBV evolution, increasing our confidence in the accuracy of the branching order. The basal dichotomy sorting out the crested newt morphotypes can be associated with a major vicariant event, but we cannot pinpoint drivers for the other two splits sorting out the morphotypes. We propose that (as yet) undiscovered vicariant events and/or ecological divergence (reflected by body shape differentiation) resulted in a disruption of gene flow. Crested newts are a suitable model to study eco-evo-devo in a rapid radiation and the new phylogenetic framework presented here serves as a baseline for future research.

## Methods

### Samples

We included seven crested newts (see Additional file 2), representing all recognized species, as well as three distinct mitochondrial DNA clades that constitute the *T. karelinii *group (cf. [16]). We follow [16] in awaiting a taxonomic revision of the *T. karelinii *group before applying specific names to the three constituent mitochondrial DNA clades (the name *T. karelinii *sensu stricto would apply to the 'eastern clade' and *T. arntzeni *has been applied to the 'western clade'; no name has as yet been proposed for the 'central clade'). We also sequenced the two marbled newts (*T. marmoratus *and *T. pygmaeus*), the remaining members of the genus *Triturus*, to function as outgroup taxa. Additionally, we added a sequence of *Calotriton asper*, sister to the genus *Triturus*, available from [31] (GenBank accession number EU880307).

### Sequences

The complete mitogenomes of the nine *Triturus *newts were sequenced in fifteen overlapping parts. We followed the laboratory protocol of [31] and designed more specific and/or internal primers where required (detailed in Additional file 3). Cycle sequencing was done commercially through Macrogen Inc. Forward and reverse sequences were checked by eye and consensus sequences were compiled with Sequencher 4.5 (Gene Codes Corporation). The fifteen fragments per individual were manually aligned and merged in MacClade 4.08 [32]. The length of the resulting sequences ranged from 16,424 to 16,649 bp. The *Triturus *mitogenomes are composed of thirteen protein-coding genes, twenty-two transfer RNA genes, two ribosomal RNA genes, the D-loop, and a non-coding region (which is highly variable in length) and gene order is identical to that found in the rest of the family Salamandridae (cf. [31]). The newly produced mitogenomic sequences have been submitted to GenBank (accession numbers are noted in Additional file 2). The 15420 bp data matrix used for phylogenetic analyses, comprising the ribosomal RNA, transfer RNA and protein-coding genes, is available from TreeBASE (study ID S11081).

### Data partitioning

We compared four different partitioning strategies for organizing the mitochondrial sequence data (cf. [33]). Each partitioning strategy treated the two ribosomal RNA genes and the concatenated transfer RNA genes as separate partitions. Differences among the partitioning strategies are based on the treatment of the protein-coding genes, dividing the protein-coding data according to: first, second and third codon position (6 partitions in total), each gene (16 partitions); first plus second and third codon position for each gene (29 partitions); and first, second and third codon position for each gene (42 partitions). The most appropriate model of sequence evolution for each data partition was determined with MrModeltest 2.2 [34], based on the Akaike Information Criterion (see Additional file 4). The optimal partitioning scheme was selected based on the differences in the harmonic mean of the -ln likelihood scores resulting from the Bayesian phylogenetic inference. The 2 ln Bayes factors were calculated for each partitioning strategy by subtracting the score resulting from simpler partitioning strategies and multiplying the outcome by -2 [35,36]. A value for 2 ln Bayes factor exceeding 10 was used as a threshold for preferring the more complex model [37].

### Phylogenetic analyses

Mixed-model Bayesian phylogenetic inference was carried out with MrBayes 3.1.2 [38]. For each data partition the rate of sequence evolution and parameters were unlinked. Two simultaneous four chain runs proceeded for one hundred million generations, with a sampling frequency of 0.001 and a heating parameter of 0.05. The first half of the sampled trees was discarded as burn-in and the phylogenetic inference was drawn from the remaining 'forest'. Tracer [39] was used to check for stabilization of overall likelihood within and convergence between runs. Partitioned Maximum Likelihood phylogenetic inference was carried out with RAxML 7.2.7 [40]. Robustness of the tree was tested via 100 bootstrap replicates. All phylogenetic analyses were carried out via the CIPRES Science Gateway [41].

### Temporal calibration

We used two independent calibration points for molecular dating, one fossil-based and one geology-based (cf. [16]). A fossil dated at 24 Ma was interpreted as a minimum estimate for the most recent common ancestor of the genus *Triturus *(cf. [42]) and the origin of the Adriatic Sea at 5.33 Ma, at the end of the Messinian Salinity Crisis, was interpreted as the vicariant event causing the *T. carnifex *- *T. macedonicus *split (cf. [6]). Divergence times were estimated with r8s 1.71 [43] and BEAST 1.5.3 [44]. In r8s we used the penalized-likelihood approach in combination with the truncated-Newton algorithm. *Calotriton asper *was pruned from the dataset, while keeping the root position, to avoid performing the time estimation on a basal trichotomy. The optimal smoothing parameter (S = 1) was determined by a cross-validation procedure, using the Bayesian consensus tree as input. Mean temporal estimates and 95% confidence intervals were determined by profiling the last thousand sampled Bayesian topologies. In BEAST, we applied the uncorrelated lognormal relaxed clock model and a Yule speciation model. The fixed calibration point was appointed a normally distributed prior with a small standard deviation (0.001) and the minimum estimate a lognormally distributed prior with the default standard deviation (1.0). The tree resulting from the Bayesian analysis was used as starting topology. Each data partition was allowed its own model of sequence evolution, as previously determined with MrModeltest. Divergence times were estimated based on two independent 100 million generation runs, sampled every 1000 generations, after discarding the first half of generations as burn-in. Tracer [39] was used to check whether effective sample sizes were at least 200.

## Authors' contributions

BW conducted laboratory work. BW and JWA analyzed the data and wrote the manuscript. All authors read and approved the final manuscript.

## Supplementary Material

Additional file 1 **NRBV tracing for the Salamandridae**. By tracing NRBV evolution over a phylogeny of the Salamandridae, the ancestral NRBV state for *Triturus *is determined. Click here for file

Additional file 2 **Sampling details**. The *Triturus *material included in this study. Click here for file

Additional file 3 **Primer information**. The primers used to sequence the *Triturus *mitochondrial genomes. Click here for file

Additional file 4 **Models of sequence evolution**. Models of sequence evolution for different data partitions as suggested by MrModeltest. Click here for file
