# Supplementary material for: Unraveling the rapid radiation of crested newts (Triturus cristatus superspecies) using complete mitogenomic sequences
Source: BMC Evol Biol. 2011 Jun 14;11:162. doi: 10.1186/1471-2148-11-162 (PMC3224112; doi:10.1186/1471-2148-11-162)
Supplement: Additional file 1 — NRBV tracing for the Salamandridae. By tracing NRBV evolution over a phylogeny of the Salamandridae, the ancestral NRBV state for Triturus is determined. [file 1471-2148-11-162-S1.PDF]

Additional file 1: NRBV tracing for the Salamandridae. By tracing NRBV evolution over a phylogeny of the Salamandridae, the ancestral NRBV state for *Triturus* is determined.

To determine the ancestral NRBV state for *Triturus*, we traced NRBV over the phylogeny of the Salamandridae family. NRBV data are taken from [1-3]. The phylogeny used is based on [4-6]. Character state tracing was done with MacClade [7]. The character state found for the most recent common ancestor of *Triturus* is ambiguous (Figure A1). It is either 12 (as in *T. marmoratus* and *T. pygmaeus*) or 13 (as in *T. karelinii* group). Higher counts in *Triturus* are derived character states. Because an NRBV character state of 13 is most regularly found in the Salamandridae (including in the earliest offshoot *Salamandrina*) we interpret 13 to be the ancestral state for the genus *Triturus*. Note that using 12 as the ancestral NRBV state instead would not influence our conclusions.

References:

1. Lanza B, Arntzen JW, Gentile E: **Vertebral numbers in the Caudata of the Western Palearctic (Amphibia)**. *Atti Mus Civ Stor Nat Trieste* 2010, **54**:3-114.
2. Chan LM, Zamudio KR, Wake DB: **Relationships of the salamandrid genera *Paramesotriton*, *Pachytriton*, and *Cynops* based on mitochondrial DNA sequences**. *Copeia* 2001, **2001**(4):997–1009.
3. Wu Y, Wang Y, Jiang K, Chen X, Hanken J: **Homoplastic evolution of external colouration in Asian stout newts (*Pachytriton*) inferred from molecular phylogeny**. *Zoologica Scripta* 2009, **39**(1):9-22.
4. Steinfartz S, Vicario S, Arntzen JW, Caccone A: **A Bayesian approach on molecules and behavior: reconsidering phylogenetic and evolutionary patterns of the Salamandridae with emphasis on *Triturus* newts**. *Journal of Experimental Zoology Part B: Molecular and Developmental Evolution* 2007, **308B**(2):139-162.
5. Zhang P, Papenfuss TJ, Wake MH, Qu LH, Wake DB: **Phylogeny and biogeography of the family Salamandridae (Amphibia: Caudata) inferred from complete mitochondrial genomes**. *Mol Phylogenet Evol* 2008, **49**(2):586-597.
6. Weisrock DW, Papenfuss TJ, Macey JR, Litvinchuk SN, Polymeni R, Ugurtas IH, Zhao E, Jowkar H, Larson A: **A molecular assessment of phylogenetic relationships and lineage accumulation rates within the family Salamandridae (Amphibia, Caudata)**. *Mol Phylogenet Evol* 2006, **41**(2):368-383.
7. Maddison DR, Maddison WP: **MacClade 4: analysis of phylogeny and character evolution, version 4.08**. *Sunderland (Massachusetts): Sinauer Associates* 2005.

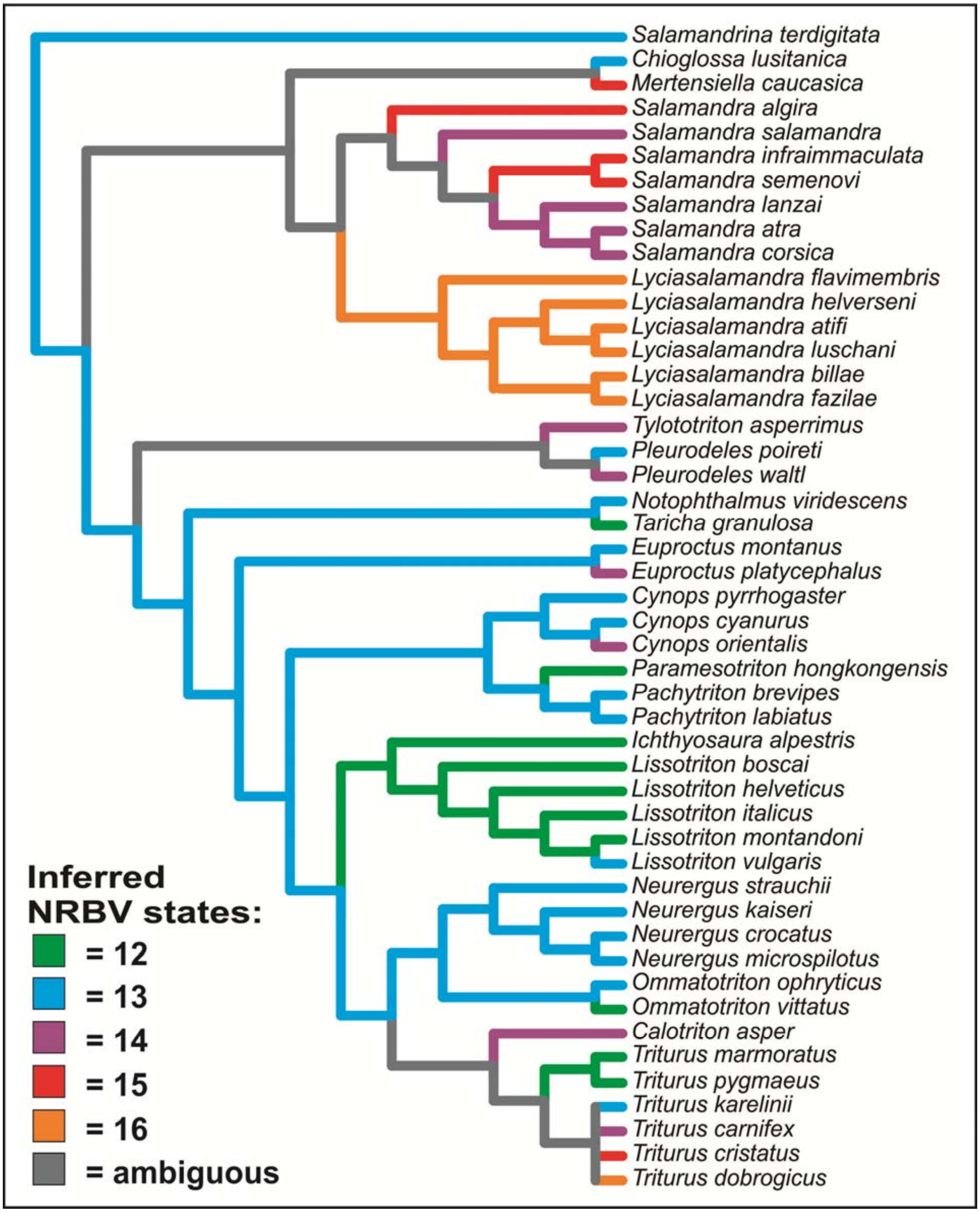

Figure A1. Determining the ancestral NRBV state for the genus *Triturus*. The ancestral NRBV state for the genus *Triturus* is deduced to be either 12 or 13 by tracing NRBV over the Salamandridae phylogeny. In *T. dobrogicus*, NRBV = 16 or 17 at roughly the same frequency, but for ease of presentation, only NRBV=16 is used.
